# Occlusal outcome after orthodontic treatment with preadjusted straight-wire and standard edgewise appliances: A retrospective cohort study

**DOI:** 10.1007/s00056-020-00273-z

**Published:** 2021-01-13

**Authors:** Spyridon N. Papageorgiou, Raphael Tilen, Vaska Vandevska-Radunovic, Theodore Eliades

**Affiliations:** 1 Clinic of Orthodontics and Pediatric Dentistry, Center of Dental Medicine, University of Zurich, Plattenstraße 11, Zurich, Switzerland; 2 Department of Orthodontics, Faculty of Dentistry, University of Oslo, Oslo, Norway

**Keywords:** Treatment outcome, Treatment duration, Orthodontics, Fixed appliances, Retrospective cohort study, Ergebnis der Behandlung, Behandlungsdauer, Kieferorthopädie, Festsitzende Apparaturen, Retrospektive Kohortenstudie

## Abstract

**Purpose:**

Orthodontic fixed appliances have been proven to be effective in treating a wide variety of malocclusions, and different types of appliances have emerged during recent decades. However, the comparative effects of different appliances have not been adequately assessed. Thus, the aim was to assess the occlusal outcome of orthodontic treatment with preadjusted straight-wire (SWIRE) and standard edgewise (SEDGE) appliances.

**Methods:**

In all, 56 patients (mean age: 13.5 years; 45% male) receiving extraction-based treatment with either SWIRE or SEDGE appliances were included. Between-group differences in the occlusal outcome assessed with the American Board of Orthodontists Objective Grading System (ABO-OGS) and treatment duration were analyzed statistically at the 5% level.

**Results:**

The average ABO-OGS score was 31.3 ± 7.2 points and 34.0 ± 10.4 points in the SWIRE and SEDGE groups with no statistically significant difference between groups (*P* = 0.26). Treatment duration was significantly shorter in the SWIRE group compared to the SEDGE group, with an average difference of −6.8 months (95% confidence interval [95% CI] = −9.6 to −4.0 months; *P* < 0.001). Likewise, fewer visits were needed with SWIRE compared to SEDGE appliances with an average difference of −7.2 visits (95% CI = −10.3 to −4.2 visits; *P* < 0.001). Adjusting for the influence of any potential confounders did not considerably impact the results.

**Conclusion:**

Similar treatment outcomes were observed after premolar extraction treatment with SWIRE and SEDGE appliances. On the other hand, SEDGE appliances were associated with prolonged treatment duration and more visits needed to complete treatment compared to SWIRE appliances.

**Supplementary Information:**

The online version of this article (10.1007/s00056-020-00273-z) contains supplementary material, which is available to authorized users.

## Abstract (de)

**Zielsetzung:**

Festsitzende kieferorthopädische Apparaturen haben sich bei der Behandlung einer Vielzahl von Fehlstellungen als wirksam erwiesen, und in den letzten Jahrzehnten sind verschiedene Apparaturtypen entwickelt worden. Die unterschiedlichen Auswirkungen der verschiedenen Apparaturen sind jedoch noch nicht angemessen erforscht worden. Ziel war es daher, das okklusale Outcome nach einer kieferorthopädischen Behandlung mit vorjustierten SWIRE(„straight-wire“)- und SEDGE(„standard edgewise“)-Apparaturen zu untersuchen.

**Methoden:**

Insgesamt wurden 56 Patienten (Durchschnittsalter 13,5 Jahre, 45% männlich) aufgenommen, die eine extraktionsbasierte Behandlung entweder mit SWIRE- oder SEDGE-Geräten erhielten. Die Unterschiede zwischen den Gruppen hinsichtlich des anhand des ABO-OGS (American Board of Orthodontists Objective Grading System) bewerteten okklusalen Outcomes und der Behandlungsdauer wurden auf der 5%-Ebene statistisch analysiert.

**Ergebnisse:**

Der durchschnittliche ABO-OGS-Score betrug 31,3 ± 7,2 Punkte in der SWIRE- und 34,0 ± 10,4 Punkte in der SEDGE-Gruppe und es gab keinen statistisch signifikanten Unterschied zwischen den Gruppen (*P* = 0,26). Die Behandlungsdauer war in der SWIRE-Gruppe im Vergleich zur SEDGE-Gruppe mit einem durchschnittlichen Unterschied von -6,8 Monaten (95% Konfidenzintervall [95%-KI] −9,6 bis −4,0 Monate; *P* < 0,001) signifikant kürzer. Entsprechend waren mit SWIRE weniger Kontrolluntersuchungen erforderlich als mit SEDGE-Geräten, mit einer durchschnittlichen Differenz von -7,2 (95%-KI -10,3 bis -4,2; *P* < 0,001). Die Anpassung unter Berücksichtigung des Einflusses möglicher Confounder hatte keinen wesentlichen Einfluss auf die Ergebnisse.

**Schlussfolgerung:**

Ähnliche Behandlungsergebnisse wurden nach Prämolarenextraktionsbehandlung mit SWIRE- und mit SEDGE-Apparaturen beobachtet. Auf der anderen Seite waren bei der Verwendung von SEDGE-Apparaturen im Vergleich zu SWIRE-Apparaturen eine längere Behandlungsdauer und mehr Besuche erforderlich, um die Behandlung abzuschließen.

## Introduction

Reliable assessment of patient records after treatment with a reliable tool is important in measuring treatment success in an objective way. Multiple indices have been introduced that make case evaluation easier [1–5] and although the Peer Assessment Rating tool [4] is one of the most widely used, it does mostly measure malocclusion improvement and not precisely measure tooth positions within the occlusion. The Index of Complexity Outcome and Need (ICON) [5] was ambitiously developed to easily evaluate case complexity, treatment need, and malocclusion improvement in an objective way, but its esthetical component might receive disproportionate weight for assessments of treatment outcome.

A more efficient method to assess outcome of orthodontic treatment might be the Objective Grading System (OGS) from the American Board of Orthodontics (ABO) [6] that uses cast models and orthopantomograms after debonding. The ABO-OGS tool gauges finishing quality of the final occlusion based on eight criteria reflecting ideal intercuspation and function. Ideal arch alignment and intercuspation are scored with 0 points, deviations from ideal for each criterion are scored with 1–2 penalty points per tooth and a case can ultimately be categorized as “success” or “failure”—a system that shows high accordance in both inter- and intraexaminer level. The ABO-OGS has been extensively used in recent years— to compare several orthodontic treatment models and techniques [7–16] with enhanced reliability, validity, and precision when evaluating the progress or final outcome of fixed appliance treatment [17, 18]. Furthermore, clinical data indicated that treatment outcomes fulfilling the standards of the ABO-OGS led to a balanced anterior temporalis activation and improved chewing function as reported by patients [19]. Finally, ABO-OGS is able to detect fine changes that occur in the occlusion postdebonding [20, 21] and evidence supports that better finishing quality with ABO-OGS leads to better long-term outcomes [22].

Fixed appliance treatment has become an integral part of modern orthodontics and has been a major focus point of orthodontic research. Since their initial introduction by Edward H. Angle, considerable development has been seen, with appliances becoming more and more sophisticated. A major historical point was the development of the preadjusted appliance by L. Andrews [2], which was followed by several variations in the values for tip and torque prescribed for each tooth. In recent years, focus has been added to try to make orthodontic treatment as efficient as possible, so as to minimize adverse effects and maximize patient satisfaction [23]. A recent systematic review on various orthodontic fixed appliances [24] concluded that at the present there is very little evidence from controlled trials to form clinical recommendations about their comparative efficacy. Comparisons between preprogrammed and nonprogrammed appliances are limited and, in their majority, suffer from methodological limitations of uncontrolled studies including confounding and assessor bias.

The aim of this study is therefore to compare the results of orthodontic treatment with preadjusted straight-wire (SWIRE) and nonprogrammed standard edgewise (SEDGE) appliances. The primary research question is whether SWIRE appliances result in better occlusal outcome measured by ABO-OGS after class II extraction treatment compared to SEDGE appliances.

## Materials and methods

### Protocol, registration, and ethical approval

The protocol for this study was developed a priori, registered in the ISRCTN registry (ID 13048456), is openly available in the Open Science Framework (https://osf.io/e3j5 f/), and deviations from it were noted (Appendix 1). Ethical approval was received from the ethical institutional authorities of the University of Zurich (BASEC no.: 2018-00631) and the University of Oslo (Regional Committees for Medical and Health Research Ethics; ref. no.: 2017/1885). This report is based on the Strengthening the Reporting of Observational Studies in Epidemiology (STROBE) statement [25].

### Sample

Included in this retrospective parallel multicenter cohort study were patients seeking comprehensive orthodontic treatment with fixed appliances with premolar extractions in two postgraduate university clinics in Oslo (Norway) and Zurich (Switzerland). As a standard procedure, informed consent was acquired from all patients or their guardians before treatment. Eligible patients should comply with the following patient-related inclusion criteria: (i) any ethnicity or race; (ii) male or female; (iii) class I, class II, or class III malocclusion; (iv) full complement of teeth excluding the third molars; (v) no previous orthodontic treatment; (vi) no dentofacial deformities or clefts; and (vii) complete set of pretreatment and posttreatment records. Additionally, they should comply with the following treatment-related inclusion criteria: (i) one phase treatment with labial fixed appliances in both arches (no two-phase treatment); (ii) bilateral extraction of a premolar in one or two jaws (either 2‑ or 4‑premolars extracted); (iii) no temporary anchorage devices of any form; (iv) no orthognathic surgery; (v) no dental trauma; and (vi) no impacted canines. Patients from the two university clinics were selected randomly from patients treated in the last 10 years that fulfilled the eligibility criteria without consideration of pretreatment characteristics, treatment outcome, or treatment duration.

Treatment planning and treatment procedures followed each institution’s typical workflow, where all patients are treated by dentists undergoing specialty training in orthodontics under the direct supervision of university faculty with extensive clinical experience. Conventionally ligated labial fixed appliances were used in all cases with the only difference that one clinic (University of Oslo) used SWIRE appliances (MBT Victory, 3M Unitek, Monrovia, CA, USA) and the other clinic (University of Zurich) used SEDGE appliances (Mini Twin Diamond, Ormco, Orange, CA, USA). Contrary to the University of Oslo, in the University of Zurich patients are primarily treated in most cases with SEDGE appliances and only a handful of selected simple cases of each postgraduate student (less than 3%) are treated with SWIRE appliances. Both appliance systems had a 0.018-inch slot and the archwire sequence/mechanics were left to the discretion of the experienced clinical instructors supervising treatment.

The records used in this study were pre- and posttreatment plaster dental casts, panoramic x‑rays, and lateral cephalometric x‑rays. These records had already been taken within the normal course of orthodontic treatment after consent of the patient for diagnostic reasons, according to the principles of evidence-based medicine and the as low as reasonably achievable (ALARA) principle. All lateral cephalometric x‑rays were taken in natural head position and were analyzed using appropriate orthodontic analysis methods.

From each patient’s documentation the following pre-treatment data were extracted: age, sex, overjet, overbite, ANB angle, and SN-ML angle. In addition, the pretreatment Discrepancy Index (DI), a pretreatment scoring system developed by the ABO for phase III of the orthodontic board certification exam, was calculated. The DI has become an accepted and reliable index for quantifying the complexity of cases based on pretreatment orthodontic record analysis and measurements from dental casts and radiographs [26, 27]. Finally, it was noted whether (i) 4 (instead of 2) premolars were extracted, (ii) a transpalatal arch was used, and (iii) a headgear was used in conjunction with the fixed appliances.

### Sample size calculation

A priori sample size calculation for the primary outcome of ABO OGS was done based on the previous study of Mislik et al. [28] using: (i) control mean of 25.7 points, (ii) standard deviation of 8.7 points—assumed common between groups, (iii) a clinically meaningful difference in ABO OGS of 30% of the control mean, (iv) use of an unpaired Student’s t‑test, (v) alpha of 5%, and (vi) beta of 20%. With these baseline data and assumptions, a needed sample of 22 patients/group (for a total of 44 patients) was calculated. In order to account for patient losses due to poor-quality radiographs/models this was rounded up by 25% to 28 patients/group (to a total of 56 patients).

### Outcomes

The primary outcome of this study was the total ABO-OGS score after debonding, measured from the patient’s posttreatment plaster models and orthopantomograms. All eight ABO-OGS components were evaluated with the special ABO gauge: alignment, marginal ridges, buccolingual inclination, overjet, occlusal contacts, occlusal relationships, interproximal contacts, and root angulation. The principal investigator (SNP) and a second author (RT) had prior to the study completed the necessary calibration process as instructed by the ABO and were calibrated with ten random cases not included in this study. The overall cumulative score for all ABO-OGS categories was used as primary outcome. The secondary outcomes included (a) the score of each separate ABO-OGS category, (b) treatment duration in months, and (c) number of visits. No blinding considering treatment decisions and procedures could have been undertaken. However, all study material was anonymized and subsequently scored, so that outcome measurement and statistical analysis could be performed in a blinded manner.

### Statistical analysis

Normality was checked through visual graph inspection and formally with the Shapiro–Wilk test. Descriptive statistics were calculated including means with standard deviations (SDs) for normal data or medians with interquartile ranges (IQRs) for non-normal data. Differences between groups were assessed with Student’s t‑tests for independent samples or Mann–Whitney tests, accordingly. Crude linear regression modelling was used to assess the effect of appliance on the primary or secondary outcome (total ABO-OGS score and treatment duration, respectively) and its 95% confidence intervals (CIs). Adjusted analyses were done using the change-in-estimate method to select potential confounders with a minimum of 10% change set as cut-off [29]. A sample of 25 patients was randomly chosen and measured by both the first (SNP) and the second author (RT), while another random sample of 25 patients was remeasured by the first assessor (SNP) after 2 weeks for repeatability. Repeatability and agreement of the measurements were assessed with the concordance correlation coefficient [30] and the Bland–Altman method [31]. Alpha was set at a two-sided *P* < 0.05, all analyses were done in Stata SE 14.2 (StataCorp, College Station, TX, USA), and the data set was openly provided [32].

## Results

A total of 56 patients were included in this study, who were treated either with SWIRE (*n* = 28) or with SEDGE appliances (*n* = 28). No significant differences existed for most characteristics (Table 1). Among the included patients 25 (45%) were male, the mean age was 13.5 years (SD = 2.4 years), the mean overjet 5.0 mm (SD = 2.2 mm), the mean overbite 3.1 mm (SD = 2.4 mm), the mean ANB 3.9° (SD = 2.2°), and the mean SN-ML 36.2° (SD = 5.6°). A total of 40 patients (71%) were treated with 4 premolar extractions and 43 patients (77%) received a transpalatal arch. The only statistically significant difference between groups was the use of headgear, where more SWIRE patients received a headgear than SEDGE patients (89% versus 64%, respectively; *P* = 0.03).

**Table 1:** Characteristics of patients included in the study Merkmale der in die Studie aufgenommenen Patienten

	SWIRE	SEDGE	*P*
Patients, *n*	28	28	–
Male, *n* (%)	14 (50%)	11 (39%)	0.42^a^
Age, mean (SD)	13.2 (1.3)	13.7 (3.2)	0.45^b^
Overjet, mean (SD)	5.1 (2.5)	4.8 (1.8)	0.60^b^
Overbite, mean (SD)	2.5 (3.0)	3.8 (1.4)	0.05^b^
ANB, mean (SD)	4.1 (2.3)	3.8 (2.2)	0.60^b^
SN-ML, mean (SD)	36.8 (5.7)	35.6 (5.4)	0.41^b^
4‑premolar extraction, *n* (%)	20 (71%)	20 (71%)	1.00^a^
TPA, *n* (%)	13 (46%)	8 (29%)	0.17^a^
Headgear, *n* (%)	25 (89%)	18 (64%)	**0.03**^a^

*SD* standard deviation, *SEDGE* standard edgewise group, *SWIRE* straight-wire group, *TPA* transpalatal arch (and/or lingual arch)

^a^ From chi square test

^b^ From t‑test for independent samples

Analysis with the DI (Table 2) showed that the two groups were on average similar in terms of baseline malocclusion severity (*P* = 0.26). The only significant differences between the two groups pertained to the DI criteria of anterior open bite and crowding. On average, patients in the SWIRE groups had significantly more anterior open bite (medians of 1.5 and 0, respectively; *P* < 0.001) and more crowding than SEDGE patients (medians of 4 and 2, respectively; *P* = 0.04).

**Table 2:** American Board of Orthodontists’ Discrepancy Index in straight-wire (SWIRE) and standard edgewise (SEDGE) groups Diskrepanzindex des American Board of Orthodontists in der SWIRE(„straight-wire“)- und der SEDGE(„standard edgewise“)-Gruppe

	SWIRE		SEDGE		*P*^a^
DI component	Median (IQR)	Range	Median (IQR)	Range	
DI category: overjet	3 (2–4)	0–8	3 (3–4)	1–7	0.45
DI category: overbite	0 (0–2)	0–5	2 (0–3)	0–3	0.25
DI category: anterior open bite	1.5 (0–4.5)	0–37	0 (0–0)	0–4	**<0.001**
DI category: lateral open bite	0 (0–0)	0–24	0 (0–0)	0–6	0.25
DI category: crowding	4 (2–7)	0–7	2 (1–7)	0–7	**0.04**
DI category: occlusal relationship	4 (0–5)	0–8	4 (1–4.5)	0–8	0.93
DI category: lingual posterior crossbite	0 (0–0)	0–2	0 (0–0)	0–4	0.79
DI category: buccal posterior crossbite	0 (0–0)	0–4	0 (0–0)	0–6	0.99
DI category: cephalometrics	3 (0–11)	0–32	4 (0–8)	0–21	0.71
DI category: other	2 (0–3)	0–6	2 (0–2)	0–8	0.91
DI total score	22.5 (14.5–31)	6–104	19.5 (15–23)	11–41	0.26

*DI* discrepancy index, *IQR* interquartile range

^a^ From Mann–Whitney test

**Fig. 1:**
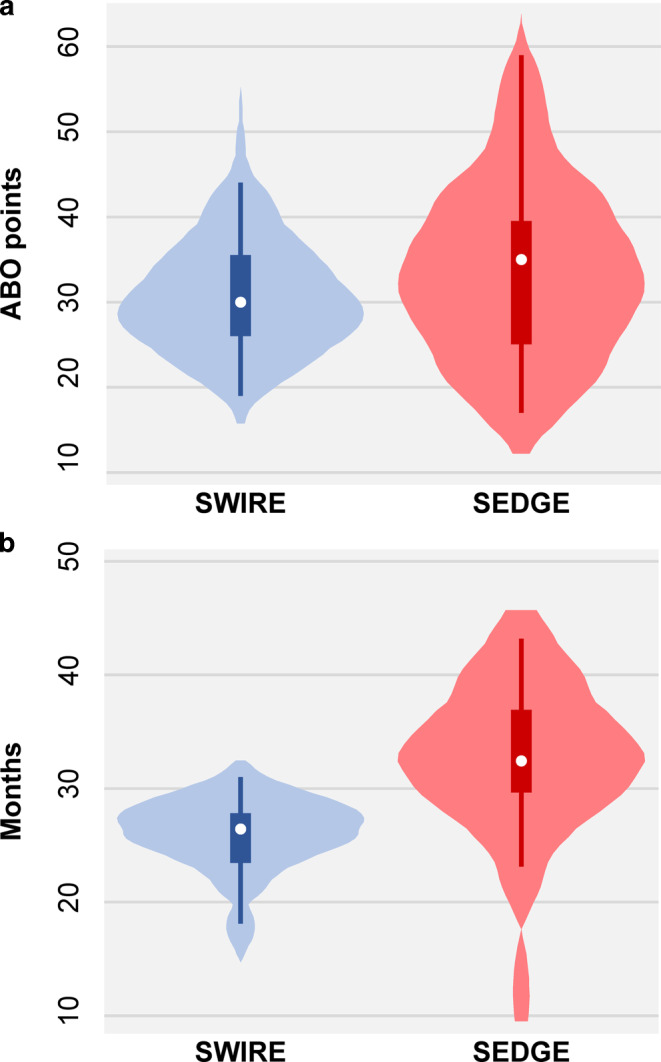
Violin plot for **a** total ABO-OGS score and **b** treatment duration in the two groups. *ABO* American Board of Orthodontics, *OGS* Objective Grading System Violin-Diagramm für **a** den globalen ABO-OGS-Score, **b** Behandlungsdauer in den beiden Gruppen. *ABO* American Board of Orthodontics, *OGS* Objective Grading System

The total ABO-OGS score was similar in the SWIRE and SEDGE groups (means of 31.3 and 34.0 points, respectively) with no statistically significant difference (*P* = 0.26; Table 3, Fig. 1a). Similar scores were also seen for most separate ABO-OGS criteria (*P* > 0.05) with the sole exception of overjet, where SWIRE patients had significantly better alignment than SEDGE patients (means of 3.9 and 5.4 points, respectively; *P* = 0.03). The treatment duration was significantly lower in the SWIRE group compared to the SEDGE group (means of 25.7 and 32.5 months, respectively; *P* < 0.001; Fig. 1b). This translated to an average difference of −6.8 months (95% CI = −9.6 to −4.0 months) between groups. The number of visits was similarly lower in the SWIRE group compared to the SEDGE group (means of 24.5 and 31.5 visits, respectively; *P* < 0.001) with an average difference of −7.2 visits (95% CI = −10.3 to −4.2 visits). This was not due to different interval between the visits (means of 4.8 and 4.5 weeks, respectively; *P* = 0.11; compared post hoc). Finally, analyses adjusted for the influence of any potential confounders (Appendix 2) found no considerable differences from the main analysis.

**Table 3:** Results of outcomes assessed Bewertungen der Behandlungsergebnisse

Variable	SWIRE	SEDGE	*P*
Patients, *n*	28	28	–
ABO Total score, mean (SD)	31.3 (7.2)	34.0 (10.4)	0.26^a^
ABO Alignment/rotations, mean (SD)	7.5 (2.4)	9.1 (3.8)	0.06^a^
ABO Marginal ridges, mean (SD)	3.7 (1.6)	4.4 (2.4)	0.19^a^
ABO Buccolingual inclination, mean (SD)	4.1 (2.8)	3.3 (2.3)	0.23^a^
ABO Overjet, mean (SD)	3.9 (2.3)	5.4 (2.9)	**0.03**^a^
ABO Occlusal contacts, median (IQR)	5.0 (3.5–6.0)	6.0 (3.0–8.0)	0.35^b^
ABO Occlusal relationships, mean (SD)	4.3 (2.0)	4.0 (2.4)	0.68^a^
ABO Interproximal contacts, median (IQR)	0 (0–0)	0 (0–0)	0.16^b^
ABO Root angulation, mean (SD)	2.3 (1.5)	1.6 (1.2)	0.06^a^
Treatment duration (months), mean (SD)	25.7 (3.5)	32.5 (6.6)	**<0.001**^a^
Number of visits, mean (SD)	24.5 (4.1)	31.5 (6.8)	**<0.001**^a^
Interval between visits (weeks) , mean (SD)	4.8 (0.5)	4.5 (0.7)	0.11^a^

*IQR* interquartile range, *SD* standard deviation, *SEDGE* standard edgewise group, *SWIRE* straight-wire group, *ABO* American Board of Orthodontists

^a^ From t‑test for independent samples

^b^ From Mann–Whitney test

Intraexaminer agreement and repeatability was almost perfect with a concordance correlation coefficient of 0.98 (95% CI = 0.96–0.99) and a Bland–Altman average difference of −0.52 (95% limits of agreement = −4.08 to 3.05). Interexaminer agreement and repeatability was somewhat worse with a concordance correlation coefficient of 0.87 (95% CI = 0.79–0.92) and a Bland–Altman average difference of −0.82 (95% limits of agreement = −9.52 to 7.87).

## Discussion

The current study assessed the occlusal outcome of 56 patients treated with premolar extractions and either SWIRE or SEDGE fixed orthodontic appliances. The main finding of this study was that the appliance type was not significantly associated with the occlusal outcome of treatment according to the total ABO-OGS score (*P* = 0.26). Small differences were seen formally only for the ABO-OGS criterion of overjet, which favored the SWIRE. It is important here to note that ‘overjet’ in the ABO-OGS pertains not to a single measurement between the upper and lower central incisors, but rather assesses the anteroposterior relationship of every pair of antagonistic anterior teeth and the buccolingual relationship of every pair of antagonistic posterior teeth. As such, differences in this criterion might reflect sagittal discrepancies of the upper to lower dentition or transverse discrepancies of the posterior tooth segments. In addition, penalties in the ‘overjet’ ABO-OGS criterion might reflect deviations from the ideal inclination or torque of any anterior or posterior tooth, which preclude proper relationships between antagonists.

On the other hand, treatment with SWIRE appliances was found to be more efficient than with SEDGE appliances in terms of shorter duration and fewer visits. This agrees with a previous retrospective study on extraction treatment that reported shorter treatment durations with SWIRE appliances [33]. A possible explanation for this might be that during space closure in the SWIRE group the already attained built-in prescription of the appliance for each tooth is retained to some degree. On the contrary, torque lost during space closure in the SEDGE group might need to be reapplied at the finishing stage, which might result in prolonged treatment times.

The problematic control of tooth inclination/torque during treatment is supported by another retrospective study of nonextraction treatment by Soltani et al. [34], where SWIRE treatment was associated with significantly improved buccolingual inclination of teeth compared to SEDGE treatment. However, a much smaller gain of 2 months in total was seen for the SWIRE group [34], but this might be due to nonextraction treatment and the lack of space closure. This difference with respect to the control of tooth inclination is corroborated by another retrospective study of extraction treatment, where SWIRE patients scored significantly better for the angulation and inclination of the maxillary posterior teeth compared to SEDGE patients [33]. Another retrospective study found significant differences in anchorage loss of the lower incisors between treatment with SWIRE and SEDGE appliances [35], which might lead to longer treatment times needed to upright proclined incisors. Finally, another study found that patients treated with SEDGE appliances did not exhibit ideal functional occlusal relationships, whereas most individuals after SWIRE appliance therapy had an ideal (i.e., mutually protected) occlusion [36]. On the other hand, an unpublished retrospective study of extraction/nonextraction cases found no statistically significant difference in either treatment duration or PAR scores between SWIRE and SEDGE appliances [37].

Although root resorption was not measured in this study, a previous retrospective study [38] reported that extraction treatment with SEDGE was associated with significantly greater resorption of the upper incisors compared to the SWIRE group. This might be attributed to more efficient force control in the SWIRE group, where tooth inclination and torque is gradually applied on the teeth already through the aligning superelastic archwires of increasing size and stiffness, whereas these are applied abruptly during the finishing stage in the SEDGE group. Finally, a previous randomized trial did not find a significant difference in either root resorption of the upper incisors or treatment duration between fully programmed (Roth) and partly programmed fixed appliances [39]. However, no pure nonprogrammed SEDGE appliance had been used in this trial, while both extraction and nonextraction cases were pooled together and this might have influenced the results.

The strengths of this retrospective observational study are its a priori registered protocol [40] and its open data provision that increases its transparency [41]. The performed sample size calculation means that the study was adequately powered to identify clinically relevant differences between appliances, while blinded outcome assessment reduces the potential for bias. However, the present study also has certain limitations that need to be considered. First, it was a nonrandomized retrospective study, which means it is more prone to bias than both prospective nonrandomized and randomized studies [42]. Furthermore, as most clinicians pick one appliance prescription and stick to it, it was not feasible to have both SWIRE and SEDGE appliances being administered in the same clinic. Thus, two different centers were compared. Pretreatment case severity was measured with the ABO DI in order to assess and control for baseline differences, but this is no panacea. Finally, important adverse effects like root resorption and gingival recessions were not assessed in this study, even though these are often taken into consideration during clinical decision-making regarding implemented appliances and techniques.

## Conclusions

The results of the present retrospective study within its limitations indicate that on average similar occlusal outcomes according to the ABO-OGS are possible after extraction-based orthodontic treatment with the use of either straight-wire or standard edgewise appliances. However, treatment with standard edgewise appliances might take longer on average than treatment with straight-wire appliances. These findings should be confirmed by future prospective clinical studies comparing these two appliances, preferably with a randomized trial design.

## Supplementary Information

Appendices: Appendix 1. Deviations from protocol. Appendix 2. Selection of covariates for the adjusted regression analyses

## Abbreviations

ABO: American Board of Orthodontics
ALARA: as low as reasonably achievable
CI: Confidence interval
ICON: Index of Complexity Outcome and Need
IQR: interquartile range
OGS: Objective Grading System
SD: standard deviation
SEDGE: standard edgewise
STROBE: Strengthening the Reporting of Observational Studies in Epidemiology
SWIRE: straight-wire
