# Supplementary material for: Occlusal outcome after orthodontic treatment with preadjusted straight-wire and standard edgewise appliances: A retrospective cohort study
Source: J Orofac Orthop. 2021 Jan 13;82(5):321–8. doi: 10.1007/s00056-020-00273-z (PMC8384812; doi:10.1007/s00056-020-00273-z)
Supplement: Supplementary file 1 — Appendices: Appendix 1. Deviations from protocol. Appendix 2. Selection of covariates for the adjusted regression analyses [file 56_2020_273_MOESM1_ESM.pdf]

APPENDICES

Appendix 1 Deviations from protocol

Section	Deviation
Outcomes	The secondary outcome of total number of archwires used within treatment was originally planned to be assessed. This was difficult to be objectively collected in a consistent way, even including an increased 28 patients per group, due to SEDGE groups manner of taking treatment notes and the use of many segmented arches / loops. Therefore, this secondary outcome was ultimately dropped.
Analysis	Initially, we had planned to include in the adjusted regression models those variables that had P ≤ 0.20 in the univariable model. However, a better variable selection method using the change-in-estimate was subsequently identified and was ultimately used.

Appendix 2 Selection of covariates for the adjusted regression analyses using the change-in-estimate method. Initially, a univariable model is constructed with the total ABO-OGS score or treatment duration as the dependent variable and fixed appliance type (standard Edgewise or straightwire) as the independent variable. Then each potential covariate is added in this initial model and the regression coefficient for appliance type on the outcome after accounting for this confounder is calculated. Finally, covariates with change-in-estimate of at least 30% are selected to be added in an adjusted analysis

	**ABO-OGS score**					**Treatment duration**			
**Adjusting for**	**β**	**Change**	**Selected**	**P**		**β**	**Change**	**Selected**	**P**
Nothing	*2.71*	-	-	-		*6.82*	-	-	-
Age	2.85	+5%	No	-		7.03	+3%	No	-
Gender	2.67	-1%	No	-		7.13	+5%	No	-
Overjet	2.77	+2%	No	-		6.74	-1%	No	-
Overbite	1.92	-29%	Yes	0.44		6.26	-8%	No	-
ANB	2.67	-1%	No	-		6.93	+2%	No	-
SN-ML	2.48	-8%	No	-		6.52	-4%	No	-
4-premolar-extraction	2.71	0%	No	-		6.82	0%	No	-
Headgear	3.10	+14%	Yes	0.21		6.87	+1%	No	-
Transpalatal arch	2.86	+6%	No	-		7.45	+9%	No	-
DI score (log)	2.86	+6%	No	-		6.75	-1%	No	-
Severe case (DI>25)	2.98	+10%	Yes	0.24		6.87	+1%	No	-

β unstandardized regression coefficient; *DI* discrepancy index

Explanation for the regression on total ABO-OGS score
Three variables with potentially confounding effect (change in estimate ≥ 10%) were found: overbite, headgear use, and case severity (DI>25). However, adjusting for these confounders still gave results similar to the original analysis and total ABO-OGS scores did not differ significantly between SWIRE and SEDGE groups (P=0.44, P=0.21, and P=0.24).

Explanation for the regression on treatment duration
No variables with potentially confounding effect (change in estimate ≥ 10%) were found. No adjusted regressions needed be run.
